# Identification of mitogen-activated protein kinases substrates in *Arabidopsis* using kinase client assay

**DOI:** 10.1080/15592324.2024.2326238

**Published:** 2024-03-17

**Authors:** Sunghwa Bahk, Nagib Ahsan, Jonguk An, Sun Ho Kim, Zakiyah Ramadany, Jong Chan Hong, Jay J. Thelen, Woo Sik Chung

**Affiliations:** a Division of Applied Life Science (BK21 Four program), Plant Molecular Biology and Biotechnology Research Center, Gyeongsang National University, Jinju, Republic of Korea; b Department of Biochemistry and Interdisciplinary Plant Group, Christopher Bond Life Sciences Center, University of Missouri, Columbia, MO, USA; c Department of Chemistry and Biochemistry, University of Oklahoma, Norman, OK, USA; d Mass Spectrometry, Proteomics and Metabolomics Core Facility, Stephenson Life Sciences Research Center, University of Oklahoma, Norman, OK, USA

**Keywords:** MPKs, Phosphorylation, Substrates, signaling

## Abstract

Mitogen-activated protein kinase (MPK) cascades are essential signal transduction components that control a variety of cellular responses in all eukaryotes. MPKs convert extracellular stimuli into cellular responses by the phosphorylation of downstream substrates. Although MPK cascades are predicted to be very complex, only limited numbers of MPK substrates have been identified in plants. Here, we used the kinase client (KiC) assay to identify novel substrates of MPK3 and MPK6. Recombinant MPK3 or MPK6 were tested against a large synthetic peptide library representing *in vivo* phosphorylation sites, and phosphorylated peptides were identified by high-resolution tandem mass spectrometry. From this screen, we identified 23 and 21 putative client peptides of MPK3 and MPK6, respectively. To verify the phosphorylation of putative client peptides, we performed *in vitro* kinase assay with recombinant fusion proteins of isolated client peptides. We found that 13 and 9 recombinant proteins were phosphorylated by MPK3 and MPK6. Among them, 11 proteins were proven to be the novel substrates of two MPKs. This study suggests that the KiC assay is a useful method to identify new substrates of MPKs.

## Introduction

Mitogen-activated protein kinase (MPK) cascades have been known to be highly conserved signal transduction modules in all eukaryotes including plants. MPK cascades are typically composed of three classes of protein kinases, MPK kinase kinase (MPKKK), MPK kinase (MPKK), and MPK. MPK cascades transduce extracellular signals to intracellular compartments to result in cellular response through the phosphorylation of downstream targets^1–3^. MPKs are activated by consecutive activation of MPKKKs and MPKKs in response to external signals. Activated MPKs directly phosphorylate the conserved motif of substrates, Ser/Thr residues followed by a Pro residue (S/T-P).^4,5^ Phosphorylation of substrates by MPKs is known to generate appropriate cellular responses by changing subcellular localization, stability, transcriptional activity, and interaction with other proteins.^6,7^

Among 20 MPKs in *Arabidopsis*, MPK3 and MPK6 have emerged as two key MPKs of primary interest because they are mainly involved in a diverse range of biotic and abiotic stresses.^8–11^ Moreover, *Arabidopsis* MPK3 and MPK6 have been shown to play important roles in plant developments, such as stomatal patterning and lateral root formation.^12–14^ In uncovering the intricate roles of MPKs in plants, the identification of new substrates of MPKs is important. To identify substrates of MPKs, several methods including protein microarray, affinity chromatography, solid-phase screening, and phosphoproteomic analysis have been attempted.^15–20^ However, it is believed that a large number of MPKs substrates have not been identified yet because the possible combinations of MPKKK-MPKK-MPK cascades are very complex, thereby the specific downstream substrates of possible combinations would be required to result in appropriate responses.

The kinase client (KiC) assay was developed as a new method for identifying the substrates of kinases through *in vitro* phosphorylation of synthetic peptide library coupled with tandem mass spectrometry.^21^ This method has potential application for high throughput characterization of kinases–substrates interaction. Successfully, 23 proteins were identified as putative substrates of 17 different protein kinases using the KiC assay.^22^ In addition, novel substrates of P2K1 (DORN1) and ILK1 were identified by the KiC assay.^23,24^ Eventually, the biological and biochemical functions of RBOHD and ILK5 isolated by the KiC assay were extensively investigated as new substrates of P2K1.^24,25^ In this study, we identified potential substrates of MPK3 and MPK6 by the KiC assay. Furthermore, these peptides were verified through *in vitro* kinase assay with recombinant proteins. As a result, we confirmed that a half of potential substrates were phosphorylated by MPKs. Finally, we identified 11 novel substrates of MPKs that would provide clues for understanding MPK-mediated signaling in *Arabidopsis*. Conclusively, this research suggests that the KiC assay is a useful tool for the identification of novel MPK substrates in plants.

## Materials and methods

### Construction of synthetic peptide library

Based on the results obtained from *in vivo* phosphoproteomic analysis in *Arabidopsis thaliana*, a library (PEPscreen, sigma, St. Louis, MO, USA) consisting of approximately 2,100 10 to 20-mer peptides was synthesized. Stock peptide solutions were prepared by dissolving the peptides in 80% (v/v) dimethylformamide in water to a final concentration of 8 mM. Samples from the stock solutions were then diluted into the KiC assay.^21,26^ These synthetic peptides were then used as the basis for an integrated experimental strategy for the identification of kinase-client proteins.

### *Expression of recombinant his-MPK3 and MPK6 in* E. coli

Full-length *MPK3* and *MPK6* cDNA were amplified by PCR from a cDNA library of *Arabidopsis* seedlings using gene-specific primers (Table S1) and subcloned into pQE30 for the expression of 6×His-fused MPK3 and MPK6. The 6×His-tag fusion proteins were expressed in *Escherichia coli* (M15) and purified using Ni-NTA agarose beads (Qiagen, Hilden, Germany).

### Phosphorylation of synthetic peptides by MPK3 and MPK6

The KiC assay was performed as previously described with slight modifications.^26^ *In vitro* kinase reaction of a mixture of synthetic peptides containing 2,100 different peptides was conducted using recombinant MPK3 or MPK6 at 37°C for 1 h with shaking. Kinase reaction was stopped by adding one volume of 1% formic acid/99% acetonitrile, evaporated to near dryness in a centrifugal evaporator, and then stored at −20°C until mass spectrometry.

### Liquid chromatography and mass spectrometry

Freeze-dried peptides were dissolved by adding 40 µL of 0.1% formic acid. Samples were loaded into 96-well plates which were then placed onto a pre-chilled 10°C auto-sampler. 10 µL of each sample was analyzed using Finnigan Surveyor liquid chromatography (LC) system attached to either a stand-alone LTQ-XL or a LTQ Orbitrap XL ETD mass spectrometer (Thermo Fisher, San Jose, CA, USA). Peptides are separated on a C18 microcapillary column using a mobile phase.

Analysis of the synthetic peptides screen was performed using a stand-alone LTQ-XL. Analysis of the recombinant protein kinase client assay was performed using a LTQ Orbitrap XL ETD. The mass spectra were collected using nanospray ionization in the positive ion mode. Detailed mass spectrometry was performed as previously described.^22^ Data-dependent ions fragment with ETD has a mass exclusion width of 10 ppm. Decision tree settings were previously described.^27^ The reagent ion source settings, including temperature, emission current, energy level, and CI pressure were 160°C, 50 µA, −70 V, and 17.5 psi, respectively. The activation time was 100 ms and supplemental activation mode was enabled.

### Bioinformatics analysis

The raw MS files were searched against a decoy database consisting of the random complement of the sequences comprising the peptide library, using SEQUEST algorithms (Proteome Discoverer 1.0, Thermo Fisher). Instrument and search parameter settings have been previously described.^22,26^ Identification data were evaluated using the XCorr function of SEQUEST, and phosphorylation-site localization was accomplished using phosphoRS (Proteome Discoverer, v. 1.0.3, Thermo Fisher). The XCorr values for each charge state were set to default and no decoy hits were allowed. Peptide mass deviation was 10 ppm and a setting of two PSMs/protein was used to further filter the data. For final validation, each spectrum was inspected manually and accepted only when the phospho-peptide has the highest pRS site probability, pRS score, XCorr value, and site-determining fragment ions allowed unambiguous localization of the phosphorylation site. Phospho-peptides with a pRS score ≥ 50 and/or a pRS site probability of ≥ 55% were accepted.

### *Expression of recombinant MPK3 and MPK6 substrate proteins in* E. coli

Full-length cDNAs of substrate were amplified by PCR from a cDNA library of *Arabidopsis* seedlings using gene-specific primers (Table S1) and cloned into the *pGEM-T Easy* Vector (Promega, USA). Verified inserts by sequencing were excised with proper restriction enzymes and subcloned into *pGEX 5X–1* for the expression of Glutathione S-transferase (GST)-fused proteins. GST-fusion proteins were expressed in *E. coli* BL21(DE3) and purified using glutathione Sepharose-4B beads (GE Healthcare, Piscataway, NJ, USA).

### In vitro *kinase assay*

*In vitro* kinase assays were performed as previously described^11^ by incubating GST-fused substrate proteins (3 μg) and 6×His-MPK3 (2 μg) or 6×His-MPK6 (2 μg) in kinase buffer (25 mM Tris-HCl, pH 7.5, 1 mM DTT, 20 mM MgCl_2_, 2 mM MnCl_2_, and 50 μM [γ-^32^P] ATP) of 20 μL. GST (1 μg; negative control) and myelin basic protein, MBP (0.5 μg; positive control) were used as negative and positive controls, respectively. The reactions were initiated using 1 μCi [γ-^32^P] ATP, allowed to proceed at 30°C for 30 min, and stopped by the addition of 4×SDS-loading buffer. Phosphorylated substrates were visualized by autoradiography after electrophoresis on 12.5% polyacrylamide gels.

## Results

### *Autophosphorylation site mapping of recombinant MPKs produced in* E. coli

**Figure 1.**
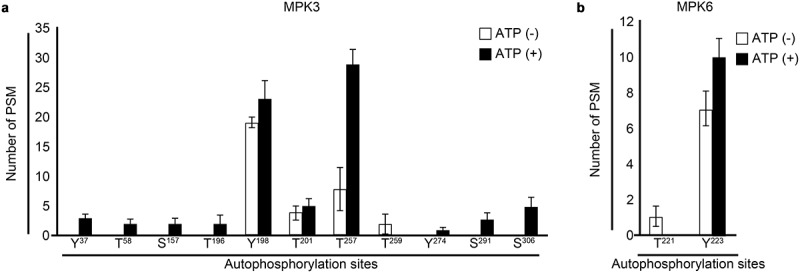
Autophosphorylation sites of recombinant MPK3 and MPK6.

MPKs are known to be fully activated by the phosphorylation of Thr-X-Tyr (T-X-Y) motif in activation loop by upstream MPKKs.^29^ Since many studies showed that recombinant MPKs produced in *E. coli* had basal activity,^9,11,15,30^ in this study, we performed the KiC assay by using recombinant MPKs purified from *E. coli* without the activation by MPKKs. Expectedly, purified recombinant MPKs had basal kinase activities. These results indicate that recombinant MPKs produced in *E. coli* may have basal activities through autophosphorylation. However, the autophosphorylation sites involved in the basal activations of MPKs are poorly identified. Therefore, we analyzed the autophosphorylation site of recombinant MPK3 and MPK6 that were produced in *E. coli* in the absence or presence of ATP. Quantitative mass spectrometry revealed that autophosphorylation occurred on multiple residues of recombinant MPKs (Figure 1). Unexpectedly, T-X-Y motif in MPK3 (Thr^196^ and Tyr^198^) and MPK6 (Thr^221^ and Tyr^223^) were already autophosphorylated before the addition of ATP. Interestingly, the autophosphorylations of Thr^196^ and Tyr^198^ residues in MPK3 and Tyr^223^ residue in MPK6 significantly enhanced by the addition of ATP (Figure 1). Furthermore, Tyr,^37^ Thr^58^, Ser^157^, Tyr^274^, Ser^291^ and Ser^406^ residues of MPK3 also were autophosphorylated only in the presence of ATP (Figure 1). This result indicates that recombinant MPKs produced in *E. coli* have their basal activities through autophosphorylation.

Quantitative mass spectrometry for identified phosphorylated sites of recombinant (a) MPK3 and (b) MPK6. Spectral count data presented are means of three replicates. Dark and white bars represent the presence or absence of ATP, respectively.

### Identification of potential substrates of MPK3 and MPK6 using the KiC assay

**Figure 2.**
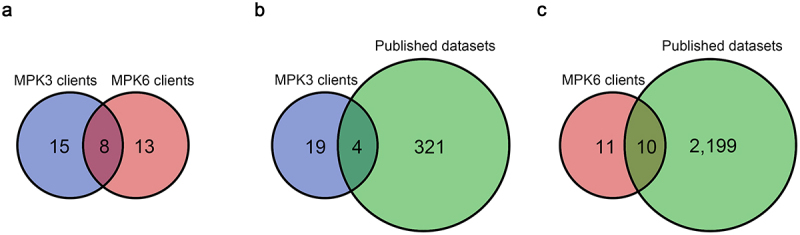
Venn diagram analysis of MPKs client peptides identified by the KiC assay.

To identify novel putative substrates of MPK3 and MPK6, we performed the KiC assay according to the standard procedure.^26^ Purified recombinant MPK3 or MPK6 was incubated with a peptide library comprised of approximately 2,100 synthetic peptides (representing *in vivo* phosphorylation events) and phosphorylated peptides were analyzed by tandem mass spectrometry. As results, we identified 23 and 21 peptides as potential client peptides of MPK3 (Table 1) and MPK6 (Table 2), respectively. Among these client peptides, eight client peptides (AT1g07630, AT1g15400, AT1g53050, AT2g38280, AT3g04470, AT4g02300, AT4g12770, and AT4g42590) were isolated by both MPK3 and MPK6, whereas 15 and 13 client peptides were specifically isolated by either MPK3 and MPK6, respectively (Figure 2A).

(a) Venn diagram showed the overlap between the identified MPK3 and MPK6 client peptides. (b) Venn diagram showed the overlap between the identified MPK3 client peptides and previously published datasets. (c) Venn diagram showed the overlap between the identified MPK6 client peptides and previously published datasets. The numbers 325 and 2,209 represent the number of reported substrates of MPK3 or MPK6 by various methods, respectively (Figure S1)^15–20^. The overlap segments indicate the numbers of the same substrates.

**Table 1.** List of identified client peptides by KiC assay using a synthetic peptide library with recombinant MPK3.

NO.	Accession	Phospho-peptide	pRS score^a^	pRS site probability (%)^b^
1	At1g01550	S*MGASTLQATSPKKAAG	143	S(1): 100.0; S(5): 0.0
2	At4g02300	T*MIMFIGDGIGKTVIKAN	135	T(1): 100.0; T(13): 0.0
3	At1g53050	RQT*QPLTSRVVTLWY	81	T(3): 98.7; T(7): 1.1
4	At1g15400	TTGRVSPAVDPPSPRIS*S*	80	S(17): 93.0; S(18): 99.4
5	At1g07630	PIVLGSGPIERGFLS*GPIER	66	S(6): 0.0; S(15): 100.0
6	At2g38280	VRPIS*PKSPVASASAF	63	S(5): 97.1; S(8): 2.7
7	At5g13240	Y*LGKSSDTDSSSPVDLLLSR	61	Y(1): 94.8; S(5): 4.9
8	At5g42590	SVSKMNSY*VSGK	59	Y(8): 98.6; S(10): 0.7
9	At3g04470	AAEEEFS*TPPSSPVFHDAK	50	S(7): 88.4; T(8): 11.2
10	At3g19330	VASTSRNDASISSPTFNLS*R	48	T(15): 0.4; S(19): 95.3
11	At3g05090	KTVFQRGGS*FLAGNLS*F	38	S(9): 99.8; S(16): 100.0
12	At4g30480	IES*SES*EDEILIKNEPK	32	S(3): 79.4; S(6): 93.4
13	At4g39680	EAQITNSATPT*T*T*PRSTGL	28	T(11): 52.1; T(12): 63.5; T(13): 59.0
14	At1g73980	LS*LDDDTVSSPKEALSRASV	25	S(2): 87.4; T(7): 3.7
15	At2g21150	GSDEDDGENKSS*GT*GNLR	23	S(12): 90.3; T(14): 90.3
16	At1g26150	ESSSPRS*DS*ALLKT*QS*SA	23	S(7): 66.1; S(9): 66.1; T(14): 66.1
17	At3g62700	MAS*PITQRSIS*IES*PR	22	S(3): 91.0; S(11): 87.7; S(14): 87.7
18	At3g01160	SSHVESEEES*ESELKVASLD	22	S(2): 47.4; S(10): 78.1
19	At4g12770	VPSSGRASVNsPTAS*QMDEL	20	S(11): 65.0; S(15): 73.0
20	At1g18670	NAS*GNKQPLT*SRVVTLW	16	S(3): 99.3; T(10): 97.7
21	At2g04235	VFARRSPEGNTNS*EIEGS*L	15	S(13): 99.8; S(18): 98.8
22	At4g18950	VKKLDDEVLS*	50	S(10): 100.0
23	At2g36570	QFELDDLLKASAEMLGKGS*	23	S(11): 24.2; S(19): 75.8

*Amino acid residues that could potentially be phosphorylated by MPK3.

^b^pRS score is based on the cumulative binomial probability that the observed match is a random event. Potential phosphorylation sites were predicted by pRS score.

^c^pRS site probabilities are estimations of the probability (0 ~ 100%) for the respective site being truly phosphorylated.

**Table 2.** List of identified client peptides by KiC assay using a synthetic peptide library with recombinant MPK6.

NO.	Accession	Phospho-peptide	pRS score^a^	pRS site probability^b^
1	At4g02300	T*MIMFIGDGIGKTVIKAN	126	T(1): 100.0; T(13): 0.0
2	At1g11360	SPTVVTVQPSS*PRFPISTPT	100	S(11): 81.5; S(17): 6.0;
3	At1g53050	RQT*QPLTSRVVTLWY	82	T(3): 99.8; T(7): 0.2
4	At3g06480	SRS*YSRSPSPVYE	63	S(1): 7.0; S(3): 85.2
5	At2g38280	VRPIS*PKSPVASASAF	59	S(5): 97.3; S(8): 2.7
6	At1g15400	TTGRVSPAVDPPSPRIS*S*	56	S(17): 93.4; S(18): 93.4
7	At2g21300	HSDDDLEEEMSPRHS*GDQSE	50	S(2): 0.5; S(11): 99.1
8	At1g07630	PIVLGS*GPIERGFLSGPIER	50	S(6): 92.7; S(15): 7.3
9	At3g55270	VHAFPLS*PTSLLRMY	47	S(7): 72.5; T(9): 12.5
10	At3g04470	AAEEEFS*TPPSSPVFHDAK	41	S(7): 90.3; T(8): 9.3
11	At5g42590	SVSKMNSY*VSGK	37	Y(8): 94.2; S(10): 2.8
12	At2g16850	AAIKALAS*FRSNPTN	37	S(8): 99.5; S(11): 0.5
13	At1g11180	GYGRTVDIPLDRPGS*GAQDL	35	T(5): 4.5; S(15): 91.1
14	At3g46450	SRNS*MMATVSSGKELLP	31	S(1): 5.7; S(4): 93.7
15	At2g41830	GLPRS*LSRTASVFSSSAALF	30	S(5): 70.4; S(7): 9.5
16	At1g08420	GALGGMVRQLS*IDQFENEGR	30	S(11): 100.0
17	At1g09770	ATRALLANYSQT*PRQGMT*P	22	T(12): 72.0; T(18): 72.0
18	At2g27060	SSTPSLPKIQNS*PDNPTS*R	22	S(12): 80.8; S(18): 71.3
19	At4g12770	VPSSGRASVNS*PTAS*QMDEL	21	S(11): 67.7; S(15): 73.7
20	At1g63730	TMLEDLPQSIRLWS*GLQV	18	S(9): 3.0; S(14): 95.4
21	At5g20280	LDVGQGLDDARS*S*PS*LLL	15	S(12): 100.0; S(13):100.0; S(15):100.0

*Amino acid residues that could potentially be phosphorylated by MPK6.

^b^pRS score is based on the cumulative binomial probability that the observed match is a random event. Potential phosphorylation sites were predicted by pRS score.

^c^pRS site probabilities are estimations of the probability (0 ~ 100%) for the respective site being truly phosphorylated.

Previously, 325 putative substrates of MPK3 and 2,209 putative substrates of MPK6 were identified through various methods such as protein microarray, primary sequence specificity, affinity chromatography and phosphoproteome analysis (Figure S1).^15–20^ To evaluate our results, we compared the isolated client peptides by the KiC assay with previously reported putative substrates of MPK3 and MPK6. As results, we found that 4 of 23 isolated client peptides (At1g15400, At2g38280, AT4g39680, and At4g12770) and 10 of 21 isolated client peptides (At1g08420, At1g09770, At1g11360, At1g15400, At2g38280, AT2g41830, At3g04470, At3g55270, At4g12770, and At5g20280) were previously reported as *bona fide* substrates of MPK3 and MPK6, respectively (Figure 2b, c).

### Nonconserved motifs are phosphorylated by MPKs in the KiC assay

**Figure 3.**
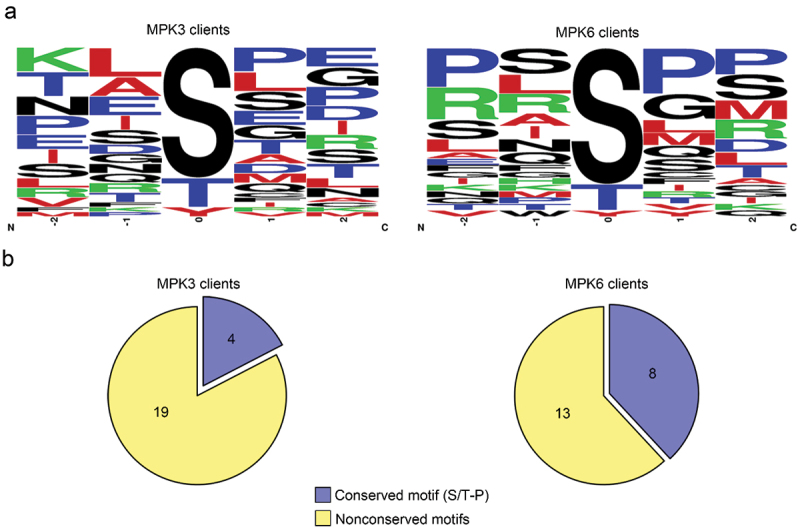
Analysis of phosphorylation sites on phosphorylated peptides.

It is well reported that MPKs mostly phosphorylate their substrates within a simple conserved motif, Ser or Thr residues followed by Pro residue (S/T-P).^4,5^ In this study, we confidently identified phosphorylation sites of isolated client peptides by tandem mass spectrometry (Tables 1,2). Almost all mapped client peptides were phosphorylated on either Ser or Thr residues. However, the Tyr residues of some client peptides were surprisingly phosphorylated by MPK3 and MPK6 (Figure 3a). Meanwhile, four and eight isolated client peptides were phosphorylated on the conserved motif by MPK3 and MPK6, respectively. However, 19 and 13 isolated client peptides were unexpectedly phosphorylated within nonconserved motifs by MPK3 and MPK6, respectively (Figure 3b). These results suggest that MPKs can phosphorylate nonconserved Ser or Thr residues and even Tyr residues in *in vitro* reconstituted experiments.

(a) Compare phosphorylated peptide sequences. The MPK3 phosphorylates on Ser (70%), Thr (24%) and Tyr (6%) residues of peptides and MPK6 phosphorylates on Ser (82%), Thr (14%), and Tyr (4%) residues of peptides. Putative phosphorylation sites were aligned on position 0. (b) Number of client peptides phosphorylated by MPKs on conserved motif or nonconserved motifs. Among 23 client peptides, 19 were phosphorylated on nonconserved motifs, whereas 4 were phosphorylated on conserved motif by MPK3. Among 21 client peptides, 13 were phosphorylated on nonconserved motifs, whereas 8 were phosphorylated on conserved motif by MPK6.

### Verification of the substrates using recombinant fusion protein

**Figure 4.**
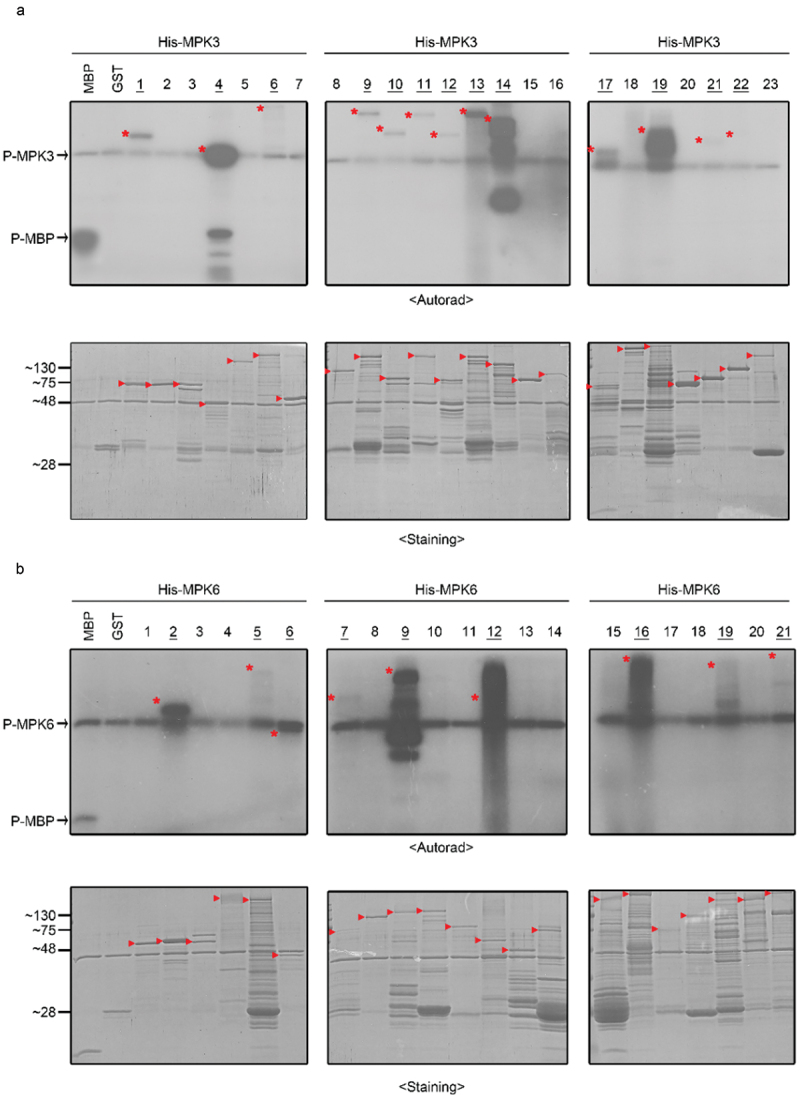
Verification of putative substrates using *in vitro* kinase assay.

Because the client peptides in the KiC assay were 10-to 20-mer short synthetic peptides, it is required to verify whether the full-length fusion proteins of isolated client peptides can also be phosphorylated by MPKs. To examine the phosphorylation of full-length proteins by *in vitro* kinase assay, we expressed and purified GST-fused full-length proteins of isolated client peptides. In the case of AT3g62700, we constructed a partial cDNA clone encoding the partial fusion protein including the potential phosphorylation site because we failed to produce its full-length fusion protein. As results, we found that 13 of 23 fusion proteins were phosphorylated by MPK3 (Figure 4A). Similarly, 9 of 21 fusion proteins were phosphorylated by MPK6 (Figure 4B). Overall, these results demonstrate that approximately half of client peptides isolated by the KiC assay were verified as the potential substrates of MPKs *in vitro*. Interestingly, 4 of 13 isolated substrate proteins have already been reported as MPK3 substrates (Table 3) and 7 of 9 isolated substrate proteins were already reported as MPK6 substrates (Table 4). Among these substrates, three substrates (AT1g15400, AT2g38280, and AT4g12770) were phosphorylated by both MPK3 and MPK6, while five substrates (AT1g08420, AT1g11360, AT3g55270, AT4g39680, and AT5g20280) were specifically phosphorylated by either MPK3 or MPK6. We compared the phosphorylation sites identified by the KiC assay with those identified in the previous reports.^15–20^ The phosphorylation sites of four substrates were same with those previously reported, but the phosphorylation sites of four other substrates were different from those previously reported (Table S2). These results indicate that not only the same phosphorylation sites but also the new phosphorylation sites can be identified from the same substrates by the KiC assay. Conclusively, based on the KiC assay we could identify nine and two novel putative substrates of MPK3 and MPK6, respectively.

*In vitro* kinase performed with (a) 6XHis-MPK3 (2 µg) or (B) 6XHis-MPK6 (2 µg) and the recombinant substrate protein (3 µg) in the presence of [γ-^32^P] ATP. After SDS-PAGE, the proteins were visualized by Coomassie blue staining (Staining). The phosphorylated proteins were visualized by autoradiography (Autorad). The asterisks represent phosphorylated substrate proteins, and the arrow represents the position of substrate proteins in SDS-PAGE. The numbers represent the fusion protein of MPK3 or MPK6 client peptides (Tables 1 and 2). MBP and GST were used as positive and negative control, respectively.

**Table 3.** List of phosphorylated recombinant substrates by MPK3 using *in vitro* kinase assay.

NO.	Accession	Annotation^a^	Phosphorylation^b^	Subcellular localization^c^	Reference
1	At1g01550	BYPASS1	++	Nucleus	
2	At4g02300	PECTIN METHYLESTERASE 39	-	Cell wall	
3	At1g53050	Protein kinase superfamily protein	-	Nucleus	
4	At1g15400	Unknown protein	++	Nucleus	Sörensson et al. 2012^17^
5	At1g07630	POL-like5	-	Nucleus	
6	At2g38280	Embryonic factor1	+	Nucleus	Hoehenwarter et al. 2013^18^
7	At5g13240	Transcription regulator	-	Nucleus	
8	At5g42590	CYP71A16	-	Extracellular	
9	At3g04470	Ankyrin repeat family protein	+	Plasma membrane	
10	At3g19330	DUF677	+	Nucleus	
11	At3g05090	Lateral root stimulator	+	Nucleus	
12	At4g30480	AtTPR1	+	Cytosol	
13	At4g39680	SAP domain-containing protein	++	Nucleus	Hoehenwarter et al. 2013^18^
14	At1g73980	Phosphoribulokinase	++	Cytosol	
15	At2g21150	XAP5	-	Nucleus	
16	At1g26150	AtPERK10	-	Plasma membrane	
17	At3g62700	ATP-binding cassette C14	+	Plasma membrane	
18	At3g01160	Unknown protein	-	Nucleus	
19	At4g12770	Chaperon DnaJ-domain superfamily protein	+	Cytosol	Hoehenwarter et al. 2013^18^
20	At1g18670	IBS1	-	Nucleus	
21	At2g04235	Unknown protein	+	Nucleus	
22	At4g18950	Integrin-linked protein kinase family	+	Nucleus	
23	At2g36570	PXC1	-	Plasma membrane	

^a^Protein annotation from TAIR database.

^b^The number of + indicates the intensity of phosphorylation by MPK3, and – indicates no phosphorylation by MPK3.

^c^Protein subcellular localization extracted from TAIR database.

**Table 4.** List of phosphorylated recombinant substrates by MPK6 using *in vitro* kinase assay.

NO.	Accession	Annotation^a^	Phosphorylation^b^	Subcellular localization^c^	Reference
1	AT4g02300	PECTIN METHYLESTERASE 39	-	Cell wall	
2	AT1g11360	Adenine nucleotide alpha hydrolases-like superfamily	++	Nucleus	Hoehenwarter et al. 2013^18^; Sörensson et al. 2012^17^; Wang et al. 2020^20^
3	AT1g53050	Protein kinase superfamily protein	-	Nucleus	
4	AT3g06480	DEAD box RNA helicase family protein	-	Nucleus	
5	AT2g38280	Embryonic factor 1	+	Nucleus	Hoehenwarter et al. 2013^18^; Wang et al. 2020^20^
6	AT1g15400	Unknown protein	+	Nucleus	Sörensson et al. 2012^17^
7	AT2g21300	ATP binding microtubule motor family protein	+	Cytosol	
8	AT1g07630	POL-LIKE 5	-	Nucleus	
9	AT3g55270	AtMKP1	++	Nucleus	Hoehenwarter et al. 2013^18^; Wang et al. 2020^20^
10	AT3g04470	Ankyrin repeat family protein	-	Plasma membrane	Wang et al. 2020^20^
11	AT5g42590	CYP71A16	-	Extracellular	
12	AT2g16850	PIP2;8	++	Cytosol	
13	AT1g11180	Secretory carrier membrane protein 2	-	Plasma membrane	
14	AT3g46450	SEC14 cytosolic factor family protein	-	Cytosol	
15	AT2g41830	Uncharacterized protein	-	Plasma membrane	
16	AT1g08420	BSL2/BRI1 suppressor 1 (BSU1)-like 2	++	Nucleus	Wang et al. 2020^20^
17	AT1g09770	AtCDC5	-	Nucleus	Wang et al. 2020^20^
18	AT2g27060	Leucine-rich repeat receptor kinase family protein	-	Plasma membrane	
19	AT4g12770	Chaperone DnaJ-domain superfamily protein	+	Cytosol	Hoehenwarter et al. 2013^18^
20	AT1g63730	Disease resistance protein (TIR-NBS-LRR class) family	-	Unknown	
21	AT5g20280	Sucrose phosphate synthase 1F	+	Plasma membrane	Wang et al. 2020^20^

^a^Protein annotation from TAIR database.

^b^The number of + indicates the intensity of phosphorylation by MPK6, and – indicates no phosphorylation by MPK6.

^c^Protein subcellular localization extracted from TAIR database.

## Discussion

### Identification of new substrates of MPKs using the KiC assay

MPK cascades in plants are believed to be composed of a large number of downstream substrates because they play a numerous variety of roles in the cellular responses to various external stimuli.^1–3^ To understand the novel functions of MPK cascades in many signaling pathways, isolation of new substrates of MPKs is essential. In this study, we applied the KiC assay to identify novel substrates of MPKs. As a result, 23 and 21 client peptides were identified as putative substrates of MPK3 and MPK6, respectively (Tables 1, 2). Interestingly, eight of identified client peptides were overlapped (Figure 2A), indicating that the targets of MPK3 and MPK6 are redundant in some signaling pathways. 13 client peptides of MPK3 and 9 client peptides of MPK6 were verified as substrates of MPKs by *in vitro* kinase assay of their fusion proteins. In conclusion, we identified nine novel substrates of MPK3 and two novel substrates of MPK6 in this study (Figure 4). Using the same KiC assay, 23 putative substrates of P2K1 were identified and some of them were extensively studied.^24^ Conclusively, the new signaling pathway of plant immune response controlled by P2K1 was elucidated by the phosphorylation studies of those isolated substrates.^24,25,32^ Thus, we suggest that the KiC assay is a useful method to identify novel substrates of MPKs and the novel signaling pathway of MPKs through these substrates may be revealed by subsequent research.

### *Validation of the KiC assay using* in vitro *kinase assay*

Generally, it is known that MPKs could phosphorylate the Ser or Thr residues on the S/T-P motif of their substrates.^4,5^ Interestingly, most isolated client peptides were phosphorylated on Ser or Thr residues, but two isolated client peptides (AT5g13240 and AT5g42590) were phosphorylated on Tyr residues by the KiC assay (Figure 3A). However, subsequent *in vitro* kinase assay of fusion proteins showed that these recombinant proteins were not phosphorylated by MPKs (Figure 4). These results indicate that the KiC assay results included nonspecific phosphorylation. Since we confirmed isolated client peptides by the KiC assay through *in vitro* kinase assay of their recombinant proteins (Figure 4), the novel substrates identified in this study would be *bona fide* substrates of MPKs.

We speculate that nonspecific phosphorylation in the KiC assay is possibly due to the excess amount of peptides and kinases employed. To improve the accuracy of the KiC assay, it is necessary to obtain results by comparing a series of different dilution reactions of peptides and kinases. Through this series of reactions can minimize nonspecific phosphorylation while maintaining efficient phosphorylation of substrates.

### Further research of novel identified MPKs-substrate modules

In this study, we identified 11 novel substrates of MPKs by *in vitro* kinase assay (Figure 4, Table 3, 4). Among them, AT1g01550 and AT3g05090 have been suggested to be involved in plant growth by participating in auxin responses,^28,33^ and AT3g73980, AT4g12700, AT4g30480, and AT2g16850 have been reported to be involved in abiotic stress response.^31,34–37^ To identify the biological roles of their phosphorylation by MPKs, it is necessary to test whether the phenotypes of these knock-out mutants can be rescued by the expression of the non-phosphorylation form. In the case of unknown substrates (AT2g04235, AT2g21300, AT3g04470, AT3g19330, and AT4g18950), the biological function of them would be identified first, and then the biological function of their phosphorylation should be investigated. In this report, we mainly focused on the usefulness of the KiC assay for the identification of novel substrates of MPKs by the assay *in vitro*. Therefore, independent more detail functional and biochemical researches including *in vivo* phosphorylation of the identified substrates by MPKs should be performed.

## Supplementary Material

Supplemental Material
