# Supplementary figures and images for: Identification of mitogen-activated protein kinases substrates in Arabidopsis using kinase client assay
Source: Plant Signal Behav. 2024 Mar 17;19(1):2326238. doi: 10.1080/15592324.2024.2326238 (PMC10950278; doi:10.1080/15592324.2024.2326238)

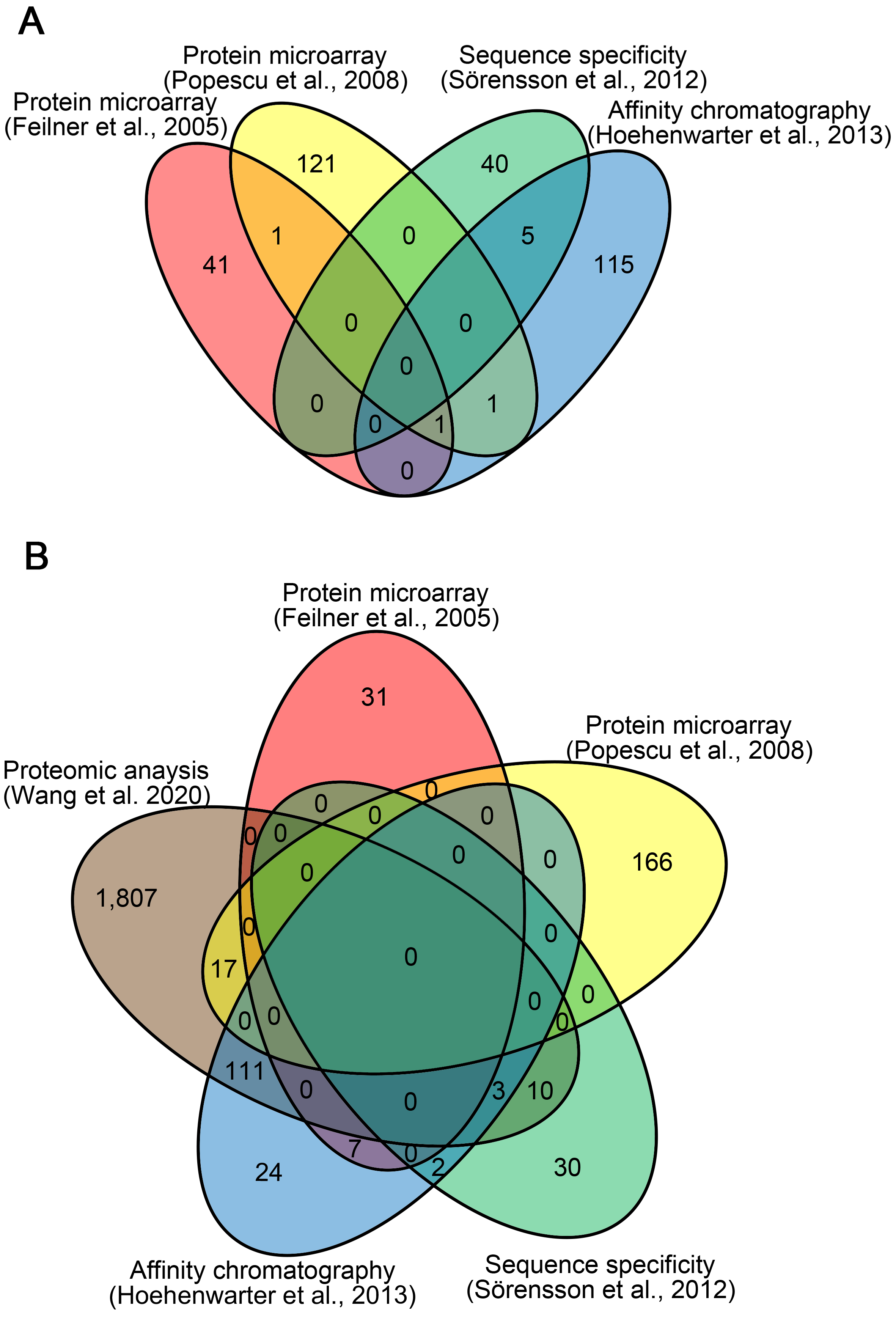

Supplement: Supplemental Material [file KPSB_A_2326238_SM7424.tif]
